# A systematic review of chiropractic care for fall prevention: rationale, state of the evidence, and recommendations for future research

**DOI:** 10.1186/s12891-022-05783-y

**Published:** 2022-09-05

**Authors:** Weronika Grabowska, Wren Burton, Matthew H. Kowalski, Robert Vining, Cynthia R. Long, Anthony Lisi, Jeffrey M. Hausdorff, Brad Manor, Dennis Muñoz-Vergara, Peter M. Wayne

**Affiliations:** 1 Brigham and Women’s Hospital and Harvard Medical School Division of Preventive Medicine, Osher Center for Integrative Medicine, 900 Commonwealth Avenue, 3rd Floor, Boston, MA 02215 USA; 2 Osher Clinical Center for Integrative Medicine, Brigham and Women’s Healthcare Center, 850 Boylston Street, Suite 422, Chestnut Hill, MA 02445 USA; 3 Palmer Center for Chiropractic Research, 1000 Brady Street, Davenport, IA 52803 USA; 4 Yale University Center for Medical Informatics, 300 George Street, Suite 501, New Haven, CT USA; 5 Center for the Study of Movement Cognition and Mobility, Tel Aviv Sourasky Medical Center, Dafna St 5, Tel Aviv-Yafo, Israel; 6 Hinda and Arthur Marcus Institute for Aging Research, 1200 Centre Street, Boston, MA 02131 USA

**Keywords:** Chiropractic, Chiropractic care, Falls, Fall prevention, Gait, Balance

## Abstract

**Background:**

Falls in older adults are a significant and growing public health concern. There are multiple risk factors associated with falls that may be addressed within the scope of chiropractic training and licensure. Few attempts have been made to summarize existing evidence on multimodal chiropractic care and fall risk mitigation. Therefore, the broad purpose of this review was to summarize this research to date.

**Body:**

Systematic review was conducted following PRISMA guidelines. Databases searched included PubMed, Embase, Cochrane Library, PEDro, and Index of Chiropractic Literature. Eligible study designs included randomized controlled trials (RCT), prospective non-randomized controlled, observational, and cross-over studies in which multimodal chiropractic care was the primary intervention and changes in gait, balance and/or falls were outcomes. Risk of bias was also assessed using the 8-item Cochrane Collaboration Tool. The original search yielded 889 articles; 21 met final eligibility including 10 RCTs. One study directly measured the frequency of falls (underpowered secondary outcome) while most studies assessed short-term measurements of gait and balance. The overall methodological quality of identified studies and findings were mixed, limiting interpretation regarding the potential impact of chiropractic care on fall risk to qualitative synthesis.

**Conclusion:**

Little high-quality research has been published to inform how multimodal chiropractic care can best address and positively influence fall prevention. We propose strategies for building an evidence base to inform the role of multimodal chiropractic care in fall prevention and outline recommendations for future research to fill current evidence gaps.

**Supplementary Information:**

The online version contains supplementary material available at 10.1186/s12891-022-05783-y.

## Background

Almost 30% of adults over 65 years of age fall each year in the United States (US) [1]. The prevalence of falls and mobility limitations increase with age with most older adults experiencing these by age 85 [2–5]. Among community-living older adults, falls are the leading cause of serious injury, disability, nursing home placement, and injury-related death [6–9]. Fatal falls in older persons exceed the death rate from the opioid epidemic by a factor of four [10, 11]. Even non-traumatic falls can lead to fear of falling, reduced physical activity, psychosocial dysfunction, and loss of autonomy [12–15].

The high incidence and long-term effects of falls among older adult results in substantial medical costs to individuals and society [16]. Falls and their consequences account for about 1–2% of all healthcare expenditures in the US, with estimates for fatal and non-fatal falls in 2015 of $637.5 million and $31.3 billion, respectively [17]. As the average age in developed countries continues to rise, both the prevalence of falls and their economic burden are expected to increase substantially [16]. This burgeoning public health crisis requires practical and effective strategies that can be readily implemented by health care providers on a national scale. The Centers for Disease Control and Prevention (CDC) recommends a multifactorial approach to fall prevention including identification of patient-specific fall risk factors, targeted exercises, and education [18]. Providers across all healthcare disciplines are being called upon to adapt to the changing needs of the older population, who are increasingly seeking alternative healthcare to address their specific needs [19, 20].

The chiropractic profession represents one of the largest health care disciplines in the world [21–23] and is utilized by an estimated 18.5% of older adults in the US [24]. Chiropractic care is provided by licensed doctors of chiropractic (DC) who focus on prevention, diagnosis, and non-pharmacological treatment of neuro-musculoskeletal conditions [25, 26]. Chiropractors have been recognized by both the American Chiropractic Association and World Federation of Chiropractic as potentially playing a key role in reducing risk of falls in aging populations [27]. Interventions included within the scope of multimodal chiropractic care are utilized by DCs dependent on factors such as individual patient diagnosis and patient treatment preferences. These interventions include passive treatments (spinal manipulation (SM) and myofascial therapies), active treatments (therapeutic exercises and mind-body interventions), or educational treatments (lifestyle modifications, self-monitoring or self-management advice) [28]. Though these nonpharmacologic therapies are used by other provider types, their use may differ substantially because factors such as specific training, practice scope, setting, and clinical skillsets likely influence how care is delivered. The benefit of these combined interventions as routinely delivered by chiropractors warrants further research.

In principle and practice, multimodal chiropractic care has the potential to mitigate many factors that contribute to fall risk in older adults including: reduced musculoskeletal strength and flexibility; chronic pain and polypharmacy; diminished proprioception and vestibular function; and overall compromised gait health, mobility, balance confidence and self-efficacy [29, 30]. The multimodal nature of chiropractic care has the potential to target multiple risk factors, and thus may afford advantages over unimodal approaches, which address a single risk factor, to managing fall risks in older adults [31, 32]. To date, little research has been devoted to evaluating the impact of multimodal chiropractic interventions on falls in older adults [33, 34]. Therefore, the broad purpose of this text is to collate research to date and to map out an evidence-based framework for studying and implementing chiropractic care strategies and programs for fall prevention in older adults. We begin by outlining a conceptual framework that includes key modifiable risk factors associated with falls in older adults, and how, in principle, components of chiropractic interventions might impact these factors. We then present methods and findings of a formal systematic review of published evidence regarding the effects of components of multimodal chiropractic care on the prevalence of falls and clinical measures of balance and gait health. Finally, based on these findings, we outline suggestions for future research to address knowledge gaps and expand the evidence-base regarding multimodal chiropractic care for fall prevention in older adults.

## Main text

### Conceptual framework for studying the impact of multimodal chiropractic care on falls, postural control, and gait health

**Fig. 1:**
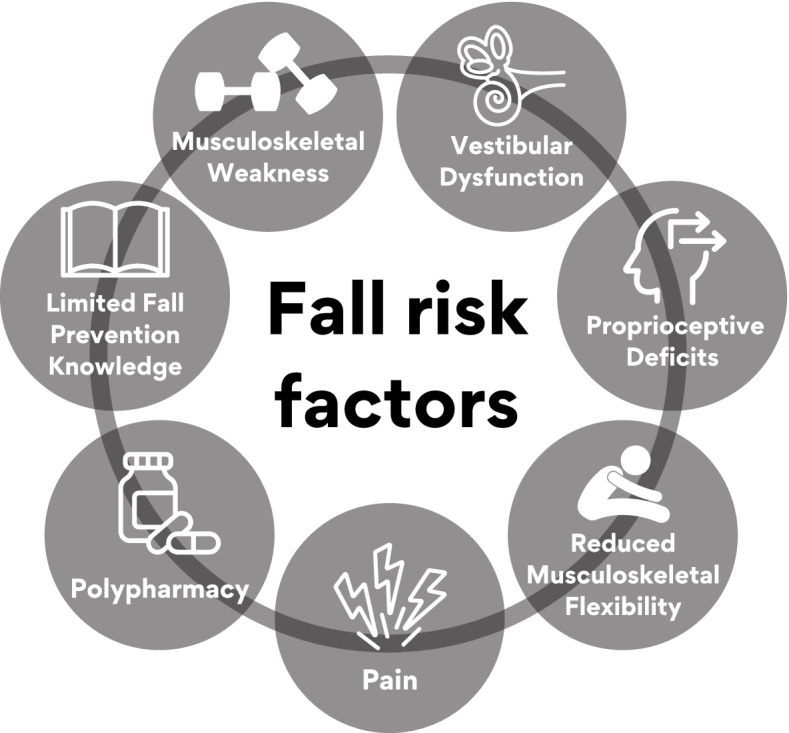
A conceptual framework summarizing fall risk factors that may be positively influenced through multimodal chiropractic care

Key modifiable factors that are targeted by multimodal chiropractic care include musculoskeletal strength and flexibility, pain, proprioception, vestibular function, and polypharmacy. In addition, knowledge gained through falls-related education could mitigate fall risks [18]. Figure 1 outlines a simplified schema of these key modifiable risk factors potentially influenced by multimodal chiropractic care. More comprehensive models that account for the complex interactions between fall risk factors are described by Shumway-Cook and Woollacott [30]. The evidence for multimodal chiropractic care influencing each of these factors is discussed below and schematically depicted in Fig. 1.

#### Musculoskeletal strength

Loss of musculoskeletal strength is a significant risk factor for falls and fall-related fractures in older adults [35, 36]. A meta-analysis of studies in older adults reported that lower extremity weakness was associated with a 70% higher likelihood for any fall and 3-fold increased risk for recurrent falls [37]. Individual studies have implicated deficits in strength in toe flexor [38], knee extension [39], and hip abductor muscles [40]. Increased fall risk is also associated with upper extremity weakness [37], and more limited evidence suggests correlations between diminished trunk muscle strength and fall risk [41]. Experimental studies have also reported that sub-normative levels of isokinetic and isometric lower extremity strength increase the likelihood of falling following an induced gait perturbation [42, 43].

Interventions commonly used in multimodal chiropractic care have the potential to positively impact musculoskeletal strength [44, 45]. These approaches can work to improve strength of targeted sets of muscles, as well as the efficiency of the overall musculoskeletal system. However, evidence that a typical course of chiropractic care can improve musculoskeletal strength, specifically in older adults, is limited. One recent randomized controlled trial (RCT) observed significant positive outcomes regarding isometric strength and core endurance in active-duty military personnel who received multimodal chiropractic care which included SM, education, advice, and reassurance [46]. Another RCT of adults with chronic non-specific neck pain, reported that a course of SM combined with exercise led to greater gains in neck strength and endurance as compared with a course of physical therapy [47]. However, parallel studies targeting chronic low back pain (LBP) did not report benefits of SM to lower extremity strength [48].

A handful of smaller studies have also evaluated the short-term impact of individual sessions of SM on changes in muscle strength. One randomized controlled crossover trial in elite Taekwondo athletes (17–50 y) reported that, compared with a passive control movement, a single session of SM increased muscle strength and corticospinal excitability to ankle plantar flexor muscles [49]. Another study in younger healthy adults (mean age 28 y) reported that lumbo-pelvic joint manipulation, compared to a lumbar passive range of motion (ROM) control, resulted in a significant increase in quadriceps force and activation immediately following intervention, but this was not sustained after 40 minutes [50]. Finally, in healthy younger adults (< 45 y) a single session of SM targeting the sacroiliac (SI) joint resulted in increased quadriceps muscle strength [51]. While the findings of these studies support a potential benefit of SM on muscle strength, because of the relatively young populations evaluated, the results are not directly applicable to older adults.

In addition to SM, other techniques commonly used as part of multimodal chiropractic care also have the potential to impact musculoskeletal strength including prescriptive or supervised rehabilitative exercise, myofascial release, and other soft-tissue techniques [52] that center largely around improving muscle tone and function. One RCT in the elderly compared the effectiveness of utilizing core stabilization exercises alone versus combined myofascial release technique and core stabilization exercise. Improvements were seen in the combined care group’s core stability endurance (measured by the supine bridge test) [53]. The utilization of exercise which targets muscular strength, balance, and gait, is widely supported in the literature as an effective component of multifactorial fall prevention strategies [31, 54]. However, as with SM, evidence regarding soft-tissue techniques is mixed [55, 56] and delivery of these interventions by chiropractors specifically has not been evaluated in older adults.

#### Musculoskeletal flexibility

Lower extremity and trunk flexibility are also associated with fall risk . In two cross-sectional studies of older adults, fallers exhibit decreased ankle dorsiflexion ROM compared to those who did not fall [57, 58]. In another cross-sectional study of older women, higher fall risk was associated with decreased trunk flexion ROM [59]. Studies in older adults have also reported that reduced extension mobility of the lower spine [60], and less flexible patterns of arm and trunk motions [61], are associated with increased fall risk.

There is evidence to support that components of multimodal chiropractic care can improve musculoskeletal flexibility, with limited studies evaluating the impact of SM alone on flexibility. One systematic review concluded that some short-term SM protocols result in clinically meaningful improvements in hamstring flexibility [62]. Another recent meta-analysis, evaluating the impact of cervical high-velocity low-amplitude manipulation reported a large positive effect on cervical ROM [45]. While links between cervical ROM and balance are not well understood, it has been suggested that in patients with neck pain there is a correlation between cervical joint stiffness, the hypertonicity of upper cervical musculature, and the presence of dizziness [63].

Techniques used by chiropractors which target the myofascial system such as Instrument Assisted Soft Tissue Manipulation (IASTM) [64], proprioceptive neuromuscular facilitation (PNF) stretching [65], and myofascial release [66, 67] may positively impact musculoskeletal flexibility. IASTM has shown benefit in multiple reviews to improve ankle dorsiflexion in both athletic and ordinary populations [64, 68, 69]. PNF stretching is known to provide short term benefits to ROM [70]. In one RCT, combined IASTM and PNF stretching showed improvements in hamstring flexibility when compared to static stretching [71]. There is limited evidence to support the success of myofascial release alone in improving ROM, but one recent systematic review reported some improvement in trunk flexibility when paired with prescriptive stretching and exercise in patients with LBP [72]. A comparative effectiveness trial in a physically active young adult population evaluated the use of myofascial release technique compared to Graston Technique (an IASTM protocol) [73]. Results showed improvements in ankle dorsiflexion with both treatments compared to a no-treatment control group [73].

#### Pain

Pain has been shown to be an independent predictor of falls [74–76]. Chronic pain is strongly associated with poor gait measures, especially while performing cognitively demanding tasks, which suggests that it may act similarly to a cognitive distraction during walking for elderly [75, 77]. Consequently, pain can contribute to difficulties with mobility and thus to an increased fall risk [57, 75, 78–80]. Pain may have additional neuromuscular effects that lead to lower extremity weakness and slower response time, caused by both lack of physical activity and reflex muscle inhibition [76, 81].

A substantiative body of research has evaluated the potential for multimodal chiropractic care to reduce or manage musculoskeletal pain. Recent systematic reviews and meta-analyses have concluded that SM as a part of multimodal chiropractic care has favorable outcomes on pain severity in patients with chronic LBP [82, 83] and acute LBP with radiculopathy [84]. Additional reviews also support that SM can be a beneficial component of treatment plans for reducing the severity of both neck [85, 86] and LBP [87, 88]. Of note, one review contrasted SM with both inactive and active (mobilization) controls. Findings were variable but indicated similar improvements in pain severity when SM was compared to the active control, and short-term improvements in pain severity when SM was compared to an inactive control [86].

Systematic reviews support the use of myofascial massage to reduce pain in the short term for chronic neck and back pain [89]. Myofascial massage has also been reported as beneficial for improving multi-site pain [90] and post-operative pain [84]. Several trials [68, 91, 92] and one systematic review [69] support the use of IASTM to reduce pain severity. Preliminary research on PNF stretching has shown improvements in pain intensity in adults with chronic LBP [93].

Exercise to reduce pain severity and disability is recognized in the literature as a crucial component of managing the severity of chronic neck and LBP [89, 94]. One systematic review reported benefits with both progressive aerobic training and progressive resistance training at reducing pain intensity [95]. Some benefit to pain management has also been noted in structured or community-based exercise programs such as yoga [96] and tai chi [94, 97].

Preliminary research also shows promise for additional therapeutic modalities used by chiropractors to manage pain including transcutaneous electrical nerve stimulation (TENS) [92] and superficial heat [84]. One randomized controlled trial reported decreased pain levels in patients with chronic LBP following a course of combined TENS and IASTM [92, 98]. Further, reviews have shown short-term benefit with utilizing superficial heat to reduce pain severity [84, 99].

#### Proprioception

Proprioception is essential for normal functioning of the body during movements, including maintaining balance and spatial awareness [36, 100]. Age-related decline in proprioception is associated with decreased functioning in muscle spindle proprioceptors, resulting in detrimental changes to sensitivity, acuity, and integration of sensory signals leading to increased risk of falls [36, 101–103]. Inhibition of input from the ankle specifically has been shown to negatively impact joint position sense [104] and altered balance [101, 105, 106]. Additional signals from proprioceptors found in the sole of the foot contribute sensory information regarding changes in distribution of contact pressures and the position of the body during stance and movement [107, 108]. Furthermore, as a result of aging and comorbid conditions, like diabetic peripheral neuropathy and lumbar nerve root impingement, sensitivity in these regions decrease and are frequently associated with a higher risk of falls [109–111].

While it has been suggested that SM can impact spinal biomechanics, paravertebral muscle activation patterns, and proprioceptor signaling [112], there is a paucity of evidence supporting the benefits of components of multimodal chiropractic care in enhancing proprioceptive function. Early research has found decreased elbow joint position sense in participants with subclinical neck pain and reported no improvement of this measure following cervical SM [113]. A more recent RCT found improvements in ankle joint position sense in community-dwelling older adults in the intervention group which received multimodal chiropractic care (variable based on provider discretion) [114]. Little research has been completed on the direct effects of SM on plantar sensation.

Components of multimodal chiropractic care such as soft tissue massage, prescriptive exercise, and kinesiology taping have shown mixed effectiveness in enhancing proprioception. Older adults who received massage following exercise saw benefit in measures of joint position sense [115] and changes in plantar sensation have been observed following the use of plantar massage [116]. The use of prescriptive exercise for the direct purpose of improving proprioceptive functioning is supported by one study showing improvement with lumbopelvic motor control exercise [117] but countered by another showing no changes following tactile acuity training [118]. A pilot study assessing the effectiveness of barefoot exercise on fall prevention in older adults found favorable effects on plantar sensation [119]. Lastly, another study reported that the use of kinesiology tape had no significant effect on lumbar repositioning errors [120].

#### Vestibular function

Along with proprioception and vision, the vestibular system is a key contributor to postural stability and control [36, 121]. Dizziness, a widespread and significant symptom of age-related vestibular hypofunction, is associated with an increased fall risk [122, 123]. Data from the National Health and Nutrition Examination Survey supports a relationship in the elderly between vestibular impairment and an increased risk of falling [124].

Evidence of chiropractic interventions to directly address vestibular dysfunction in the elderly is limited, but there is some which suggests procedures used within chiropractic may be effective for managing symptoms of dizziness. A small randomized trial evaluating the effectiveness of instrument assisted cervical or thoracic manipulation plus multimodal chiropractic care (manual soft-tissue therapy, mobilization, prescriptive exercise, and advice) reported favorable trends toward improvement measured with the Dizziness Handicap Inventory (DHI) [63]. More broadly, systematic reviews focused on treatment of cervicogenic dizziness conclude that there is moderate evidence to support the use of manual therapies, including spinal mobilization and manipulation [125, 126].

Some studies support a combination of other interventions which may be employed by chiropractors, for example physical (repositioning) maneuvers and exercise-based vestibular rehabilitation for longer-term functional recovery, including decreased levels of dizziness [127]. One study reported that the Epley maneuver was effective for long-term reduction of nystagmus among people with Benign Paroxysmal Positional Vertigo (BPPV) [128]. Others showcase chiropractic clinical utilization of the Epley maneuver for BPPV with positive results [129, 130]. Additional evidence supports that movement-based interventions, which can be prescribed by a DC as part of a treatment plan, including vestibular rehabilitation exercises and Tai Chi can improve dizziness and balance resulting from vestibular dysfunction [127, 131, 132].

#### Polypharmacy

Polypharmacy, which entails the use of multiple medications at the same time, is yet another independent fall risk factor in the elderly [133–135]. Several studies have shown the impact of polypharmacy on increasing fall risk in elderly populations, with some suggesting a monotonic dose-response relationship between the number of prescription drugs and the ensuing risk of injurious falls [136–138]. Polypharmacy increases risk of falls through adverse events such as dizziness and reduced cognitive function [139]. Opioid pain medications have been shown to either contribute directly to these symptoms, or increase the likelihood of their occurrence through drug-drug interactions [140].

Although the vast majority of chiropractors are not trained or licensed to prescribe medications, through the positive impact of non-pharmacologic interventions on pain, it is plausible that chiropractic care can facilitate reduced pain medication use. A recent retrospective study utilizing medical claims data reported patients who saw a chiropractor for their spinal pain had half the risk of filling a prescription for opioids [141]. Others have found promising negative correlations between the use of chiropractic care and opioid prescription in both the private sector and the United States Department of Veterans Affairs (VA) [142, 143]. Further, a recent large-scale observational study found that patients who had an initial visit for LBP with a chiropractor had 90% lower risk of using opioids in the short- and long-term, compared to those who sought their initial care from a primary care physician [144]. A recent systematic review and meta-analysis of observational studies in multidisciplinary healthcare systems reported that, compared to patients who do not receive chiropractic care for non-cancer pain, patients who do receive chiropractic are 64% less likely to be prescribed an opioid [145].

While direct research evaluating the impact of utilizing specific chiropractic modalities on general medication use in the older population is scarce, early trials have assessed medication use as a secondary outcome measure. One study found significant decreases in the duration of medication use in an elderly population after receiving SM and completing prescriptive home exercises [48]. The same team studied adolescent populations, comparing interventions of exercise therapy alone or a combination of SM and exercise therapy, reporting an 80% decrease in medication use for both interventions [146]. Longer-term trials of prescriptive exercise have reported small but beneficial trends toward reducing medication use over time [147, 148].

#### Education

Patient-centered education efforts have been acknowledged by the CDC as an essential part of fall prevention [18]. The efficacy of a multifactorial educational fall prevention program showed positive outcomes regarding functional improvements of an elderly population [149]. Several studies have shown benefit in reducing hospital-based falls by administering educational videos to both patients and families [150, 151]. The inherent multifactorial nature of education enhances its applicability in multiple settings by utilizing techniques such as structured health education classes, brochures, or home hazards evaluation.

Chiropractors are well-positioned to provide educational fall prevention efforts, as older patients are increasingly selecting manipulative therapies [20], and chiropractors frequently witness the outcomes of falls in their older patients [152]. One study suggests an important role of chiropractors in fall prevention may be in providing appropriate referral to patients with a history of falls [153]. Through evaluation of gait patterns they can also advise on footwear or determine if a podiatric intervention is necessary [153]. Other studies support a more active role of chiropractors as patient educators, offering advice to address environmental fall hazards in the home such as poor lighting, uneven or torn carpets, slippery floors and unsteady stationary objects [18]. However, the effectiveness of fall prevention-related education delivered by chiropractors has not been well studied.

In summary, an incomplete but growing and compelling body of studies support the concept that multiple components of chiropractic care, both independently and together, may positively impact key risk factors for falls in older adults. Below we present the findings from a formal systematic review of published studies evaluating the effect of chiropractic care on fall prevalence, as well as its impact on clinical measures of balance and gait associated with fall risk.

### Systematic review of the impact of chiropractic care on falls, postural control, and gait health

The primary goal of this systematic review, embedded within this broader narrative review, is to identify and synthesize studies to date that evaluated the impact of components of multimodal chiropractic care on prevalence of falls. Given the limited evidence on this topic, and the well-established link between clinical measures of balance and gait and fall risk [29, 154, 155], we also include studies that have assessed the impact of chiropractic care on balance and gait related outcomes. Specific components of the chiropractic scope of practice assessed in this review include SM, myofascial therapies, thermal modalities (hot and cold packs), and prescriptive rehabilitative exercises. To be comprehensive, our search was neither limited to a specific timeframe, nor by population, medical conditions, or intervention duration.

## Methods

### Literature search

Electronic literature searches were performed using PubMed, Embase, Cochrane Library, Physiotherapy Evidence Database (PEDro), and Index to Chiropractic Literature (ICL). The search was limited to the English language, and publication dates ranged from database inception to June 27, 2022. The search terms included, for example, “chiropractic,” “chiro*,” “manipulation” in combination with “gait,” “falls,” “vestibul*”, “dizz*”, or “balance.” Additional manual searches, based on references listed in the retrieved articles, were performed to complete the search. The full list of search terms is detailed in Table 1.

**Table 1:** Databases included in electronic literature search and search terms used for each

Literature Searched	Search Terms
PubMed	(chiropractic [Mesh] OR chiro*[Mesh] OR chiropractic [tiab] OR chiro* [tiab] OR “chiro* manipulation”) AND (gait [Mesh] OR gait [tiab] OR fall [Mesh] OR fall [tiab] OR balance [mesh] OR balance [tiab] OR vestibul* OR dizz*)
Embase	(‘chiropractic’/exp. OR chiropractic OR chiro OR chiropractice OR ‘chiropractice care’ OR (chiropractice AND (‘care’/exp. OR care))) AND (‘gait’/exp. OR gait OR ‘exp gait’ OR (exp AND (‘gait’/exp. OR gait)) OR ‘falling’/exp. OR falling OR ‘balance’/exp. OR balance OR ‘exp balance’ OR (exp AND (‘balance’/exp. OR balance)) OR vestibular OR ‘dizz*or dizzy’ OR (dizz*or AND dizzy)) AND (‘Article’/it OR ‘Article in Press’/it)
Cochrane Library	(chiropractic or chiro):ti,ab,kw AND (gait or fall or balance or vestibular):ti,ab,kw
PEDro	1. chiro* AND gait
	2. chiro* AND fall*
	3. chiro* AND balance
	4. chiro* AND vestibular
	5. chiro* AND dizz*
ICL	All Fields:chiro* AND All Fields:gait OR All Fields:fall* OR All Fields:balance OR All Fields:vestibular OR All Fields:dizz*, Publication Type:Clinical Trial OR All Fields:chiro* AND All Fields:gait OR All Fields:fall* OR All Fields:balance OR All Fields:vestibular OR All Fields:dizz*, Publication Type:Randomized Controlled Trial OR All Fields:chiro* AND All Fields:gait OR All Fields:fall* OR All Fields:balance OR All Fields:vestibular OR All Fields:dizz*, Publication Type:Controlled Clinical Trial

### Eligibility criteria and synthesis

RCTs, prospective non-randomized controlled trials, observational single arm clinical trials, and crossover studies published in English, in which chiropractic manipulation or multimodal care was the primary intervention and the changes in falls, clinical measures of gait, balance and balance related outcomes (e.g., vestibular function, proprioception, dizziness) were the outcomes measured. Due to the great heterogeneity and small sample sizes of the identified studies meta-analysis was not employed. Synthesis was limited to narrative summaries.

### Study selection and data extraction

Study eligibility assessment was performed independently by three researchers (WG, DMV, and WB) who applied eligibility criteria using an agreed upon protocol. Data were extracted by three reviewers (WG, DMV, and WB) independently using a standardized template generated in Microsoft Excel. Data related to study design, duration and frequency of the intervention program, type of the control group, sample size, and outcome measures were extracted for qualitative analysis in accordance with the Preferred Reporting Items for Systematic Reviews and Meta-Analyses checklists (PRISMA) and are reported in Additional Files 1 and 2 [156].

### Risk of bias assessment

Three researchers (WG, DMV, and WB) independently assessed the methodological quality of 13 RCTs and randomized crossover studies, using the updated 8 item Cochrane Collaboration Tool for assessing risk of bias [157]. The assessed criteria which included: random sequence generation, allocation concealment, blinding of participants and personnel, blinding of outcome assessment for self-reported and objective measures, incomplete outcome data (attrition rates or intention to treat), selective reporting, and other bias (e.g., carry-over effects in cross-over trials or recruitment bias in randomized controlled trials) [157]. The evaluated domains were assessed as low risk of bias (“+”), unclear risk of bias (“?”), high risk of bias (“-“), or, in certain cases, not applicable (“N/A”) according to the established criteria [58]. Any discrepancies in the evaluations conducted by two authors were discussed, and when need, resolved with the input of a fourth evaluator (PMW).

## Results

**Fig. 2:**
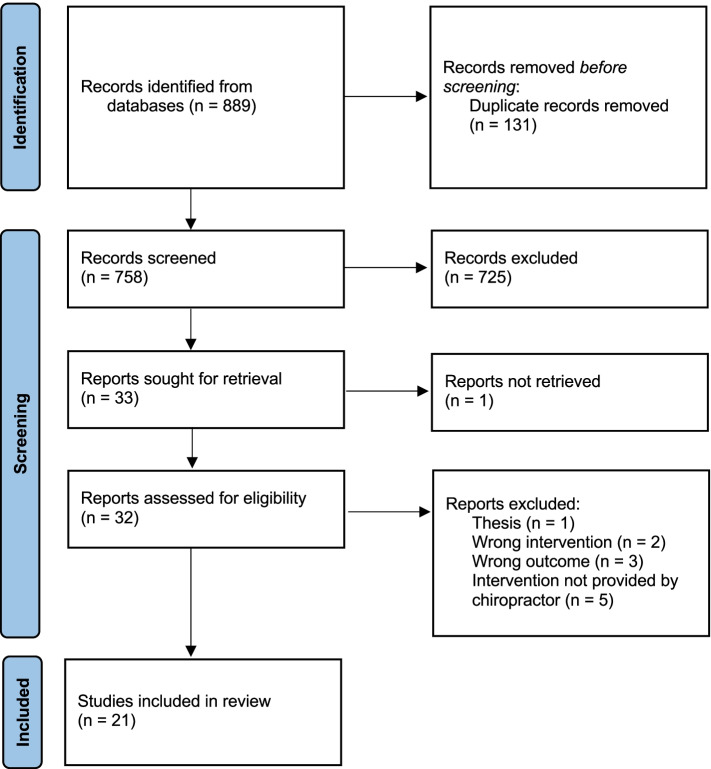
Preferred Reporting Items for Systematic Reviews and Meta-Analysis (PRISMA) flow chart

Figure 2 summarizes the flow of the literature search and selection process following PRISMA guidelines. An initial search identified 889 records from multiple databases and manual searches. Removing duplicates resulted in 758 records. The title and abstracts of these records were screened according to the inclusion criteria. A total of 32 full text articles met the initial eligibility criteria and were further reviewed. An additional 11 studies were excluded for no chiropractic intervention (*n* = 2), the intervention not being delivered by a chiropractor (*n* = 5), not reporting the outcomes of interest (*n* = 3), and a thesis (*n* = 1). Of the remaining 21 eligible studies, 10 were RCTs [27, 46, 63, 114, 158–163], 6 were single arm clinical trials [164–169], and 3 were cross-over studies [170–172]. One case series [173], and one observational study [34] were included.

Seven of the studies were purely descriptive and did not include inferential statistics [34, 63, 158, 159, 164–166]. Fourteen of the included studies utilized inferential statistics, and reported *p*-values are included, when possible, in the summary of main findings [46, 114, 160–163, 167–174]. Only three prospective studies had sample sizes greater than 100 [46, 161, 174]. The remainder had small sample sizes (< 32 per group) and thus were likely underpowered for formal inferential statistical analyses [114, 160, 162, 163, 167–173]. Three of these smaller studies included a power calculation completed a priori [114, 170, 172] and one was completed as part of the post hoc analysis [162]. The remaining studies did not complete power calculations [160, 163, 167–169, 171, 173].

### Participants’ characteristics and study settings

Table 2 summarizes the 21 included studies, reporting the most relevant findings pertaining to this systematic review. A total of 976 subjects were included. Data on baseline demographic characteristics regarding age were not reported in three studies [167–169], two studies reported median age of participants, and thus were not included in calculation of average participant age [164, 166]. Of the remaining 16 studies (*n* = 909), the average participant age was approximately 52.25 years. Two studies did not report gender distribution [168, 169] and of the remaining studies (*n* = 965) approximately 48% of participants were female and 52% were male. Two of the 21 studies recruited young and healthy adults (20–40 years of age) [171, 172], and 8 studies included older adults (60+ years of age) [34, 63, 114, 158, 159, 161, 165, 174]. Five studies enrolled participants with SI joint conditions [160, 167–169, 173], 4 included subjects with neck pain or vertigo [63, 161, 164, 166], and 2 focused on subjects with LBP [46, 169]. No studies specifically included populations at high risk of falling.

**Table 2:** Study details of articles that met final inclusion criteria (*n* = 21)

Main Author, Year (country)	Type of study	Sample (mean age)	Study Population	Intervention	Intervention: Frequency /duration	Control group	Control: Frequency/ duration	Measured outcomes	Relevant Findings
Bracher, 2000 (Brazil) [164]	SACT	15 (41 median)	Adults (27–82), in otorhinolaryngology practice, dizziness and diagnosis of cervical vertigo	Multimodal chiropractic (spinal manipulation, manual techniques, electrotherapy, medication (sedation), biofeedback, exercise)	Individual, as needed, mean 5 (3–10)	–	–	Vertigo severity, Musculo-skeletal Pain	Descriptive statistics reported from baseline to study completion: • 9 patients (60%) reported complete remission of vertigo symptoms. • 3 patients (20%) reported improved vertigo symptoms.
Hawk, 2007 (USA) [158]	RCT	11 (73.0)	Older adults (60+), OLST < 5 sec, ambulatory, no balance exercises	Multimodal (spinal manipulation, soft tissue and myofascial release, heat)	2x weekly, over 8 weeks (16 sessions total)	8 balance exercises	2x weekly, over 8 weeks (16 sessions total)	BBS, OLST, PDI, DHI, self-reported falls	Descriptive statistics reported little detectable change from baseline to study completion in the collected outcomes of intervention group: • Change in DHI scores ranged: − 12 to 32. • Change in BBS scores ranged: − 5 to 16. • Change in OLST scores ranged: − 5 to 0. • No trends observed in collected falls data.
Hawk, 2009 (USA) [159, 165]	SACT	14 (77.0)	Older adults (60+), OLST < 5 sec, ambulatory, no recent SM	Multimodal chiropractic, (HVLA, other manipulations, soft tissue treatment, hot packs)	2x weekly over 8 weeks (16 sessions total)	–	–	SF-BBS, OLST, PDI, DHI, GDS	Descriptive statistics reported from baseline to study completion: • 3/6 patients with baseline DHI scores indicating dizziness, showed clinically significant reduction (> 18 points). • Little to no trends observed in both SF-BBS and OLST scores.
Hawk 2009 (USA) [159, 165]	RCT	34 (80)	Older adults (60+), OLST < 5 sec, ambulatory, no balance exercises	Multimodal chiropractic (spinal manipulation, soft tissue and myofascial release, heat; hip, knee ankle)	GR1: 2x weekly, over 8 weeks (16 sessions total) GR2: 2x weekly, over 8 weeks + 10 monthly visits, over 10 months (26 sessions total)	GR3: Home-based balance exercises	Over the study period, no established frequency	BBS, OLST, PDI, DHI, GDS, self-reported falls (in clinical notes)	Descriptive findings reported from baseline to study completion: • Trend toward increasing BBS scores in GR2. • DHI scores improved in GR1 and GR2. • Reporting of falls was not equal among groups (GR1: 18 visits, 6 reported falls. GR2: 26 visits, 9 reported falls. GR3: 5 visits, 0 reported falls).
Herzog, 1988 (Canada) [167]	SACT	11 (−-)	Adults with unilaterally decreased mobility in sacroiliac joint	Spinal manipulation of sacroiliac joint	6 sessions over 2 weeks	–	–	Gait Symmetry, ^a^VAS, ODI, palpation-based joint mobility	Reported observations from baseline to study completion: • Improvements in symmetry observed in ML GRF between the involved and noninvolved sides. • No detected differences for vertical or AP GRF.
Herzog, 1989 (Canada) [168]	SACT	11 (−-)	Chiropractic patients with sacroiliac problems	Spinal manipulation of sacroiliac joint	Single session	–	–	Gait Symmetry^1^	• No changes observed from baseline to study completion in measures of gait symmetry or GRF.
Herzog, 1991 (Canada) [160]	RCT	37 (33.5)	Adults (18–50), ambulatory with chronic sacroiliac joint problems, nor obese	Spinal manipulation of sacroiliac joint	10 sessions over 4 weeks	Back school therapy program by PT (stretching, strengthening exercises, no manipulation)	10 sessions over 4 weeks	VAS, ODI, Gait Symmetry^1^	• SM group showed improvements in gait symmetry (in all GRF components) from baseline to study completion. • Back school therapy did not show improvement in gait measures.
Holt, 2011 (New Zealand) [34]	Observational	101 (72.0)	Older adults (65+), ambulatory, active in chiropractic care	Multimodal chiropractic care	Individual, as needed	–	–	History of falls, BBS, ABCs, Posturography^b^	Descriptive statistics reported: • 34.6% of the participants reported at least 1 fall in the prior year. • Mean BBS scores of 51.9 (SD 5.9) reported. • 59.4% of participants exhibited posturographic measures categorized as severely or profoundly impaired or were unable to complete posturographic assessment (included in profound category).
Holt, 2016 (New Zealand) [114]	RCT	60 (72.2)	Older adults (65+), community dwelling, ambulatory	Multimodal chiropractic care chiropractic (HVLA, table and instruments adjustments)	Individual, as needed (range: 2–33)	Usual care (as prior to the study)	–	Joint position, stepping reaction time, static postural control^2^, SF-36, multisensory processing^c^	• Chiropractic treatment group showed improvements in choice stepping reaction time (*p* < 0.05) and in ankle joint position sense (*p* < 0.05) compared to usual care group.
Kendall, 2018 (Australia) [63]	RCT	22 (73)	Older adults (65–85) with neck pain and concomitant dizziness > 3 months	Activator II instrument assisted manipulation with joint mobilization, massage, ROM neck exercise or heat	1x weekly, over 4 weeks (4 sessions total)	Sham intervention (Activator II instrument impulses (set at zero) and gentle placement of practitioner’s hands on the cervical and thoracic spine	1x weekly, over 4 weeks (4 sessions total)	DHI, TUG, NDI, NRS, FES-I	Descriptive statistics reported [mean (SD)] from baseline to study completion: • Improvements in DHI scores for both intervention [40.77 (12.48) to 28.33 (14.37)] and control groups [44.00 (16.97) to 36.40 (20.11)]. • Small improvements seen TUG test score in the intervention group [12.18 (2.07) to 11.87 (3.67)] but not in control group [12.09 (2.87) to 12.36 (411)].
Maiers, 2014 (USA) [161]	RCT	241 (71.7)	Older adults (65+) with neck pain, ambulatory, stable medications, MMSE score > = 20	GR1: SM with home exercise, GR2: SRE with home exercise	SM: Individualized (range 5–19) Supervised rehabilitative exercise: 20, 1-hour sessions over 12 weeks	GR3: Home exercise	4x weekly for 45–60 minutes over 12 weeks (48 sessions total)	NRS, NDI, SF-36, satisfaction, global improvement, medication use, ROM, strength, TUG	• Change in TUG test time was reported (Mean [95% CI]) as week 12 score minus baseline score, no significant between group differences reported: • GR1: (−0.3 [− 0.8 to 0.2]). • GR2: (− 0.3 [− 0.7 to 0.1]). • GR3: (− 0.2 [− 0.7 to 0.3]).
Maiers, 2019 (USA) [174]	RCT	182 (71.1)	Older adults (65+), ambulatory, community dwelling, self-reported back and neck disability > = 12 weeks	SM (HVLA, soft tissue, thermal therapy, stretching) + SRE	GR1: 12 weeks of SM as needed + 1 hour SRE session 2x in 1st month, then 1x/month GR2: 36 weeks of SM as needed + 1 hour SRE session 2x in 1st month, then 1x/month	–	–	Incidence of falls, ODI, NDI, NRS, EQ-5D, TSK, medication use, perceived improvement, self-efficacy, satisfaction, strength, SPPB, accelerometry	Incidence of falls measured through proportions and limited statistical analysis: • GR1: Proportion of falls ranged from 6 to 13%. • GR2: Proportion of falls ranged from 10 to 13%. • Between group differences at each measurement were reported as: *p* = 0.4 at 12 weeks, *p* = 0.4 at 24 weeks, *p* = 0.7 at 36 weeks, *p* = 0.8 at 52 weeks, *p* = 0.3 at 78 weeks.
Malaya 2020 (USA) [171]	Crossover (RCT)	24 (29.5)	Healthy adults (21–40), not pregnant, no major injury to the extremities, no previous surgery, no known neurological or systemic disease	GR1: Lower extremity manipulations on day 1 and upper extremity manipulations on day 2 GR2: Upper extremity manipulations on day 1 and lower extremity manipulations on day 2	Single intervention of nonspecific long-axis distractions to lower extremity (ankle, knee, and hip) or upper extremity (shoulder, elbow, and wrist)	–	–	Static postural assessment^d^	• No significant changes in pathlength or range of sway for the floor surface condition at any sensor location after manipulation. • Lower extremity manipulation affected sway dynamics of the trunk for the floor surface condition • Significant results reported for the AP rocker board surface condition after upper extremity manipulation at the trunk sensor (path *p* < 0.05 main effect; range *p* < 0.05 interaction effect) and surface sensor (path *p* < 0.05 main effect) • Significant main effect results reported for the AP rocker board surface condition after lower extremity manipulation at the trunk sensor (SampEn *p* < 0.05 interaction effect)
Malaya 2021 (USA) [172]	Crossover (RCT)	23 (27.4)	Healthy adults (21–35), not pregnant, no known musculoskeletal, neurologic or visual impairment	GR1: Lower extremity manipulations on day 1 and upper extremity manipulations on day 2 GR2: Upper extremity manipulations on day 1 and lower extremity manipulations on day 2	Single intervention of nonspecific long-axis distractions to lower extremity (ankle, knee, and hip) or upper extremity (shoulder, elbow, and wrist)	–	–	Static postural assessment^4^, COP	• Reduction in ML COP pathlength (*p* = 0.005) observed after both upper and lower extremity manipulation. • No significant change observed for range or SampEn in either group.
Osterbauer, 1993 (USA) [173]	Case series	10 (38.0)	Adults with chronic, phase 1 SIJ syndrome	Spinal manipulation (mechanical force, manually assisted, short lever adjustments)	3x weekly, over 5 weeks, 1 year follow up as needed	–	–	Slow-walking gait symmetry^1^, VAS, ODI	• No changes observed from baseline to study completion in gait symmetry or GRF.
Palmgren, 2009 (Sweden) [170]	Crossover (Time Series)	6 (34.67)	Healthy adults (28–45)	GR1: Facet nerve block then late SM to C5/C6 GR2: Early SM to C5/C6 then facet nerve block	Single manipulation/single nerve block	–	–	Posturography^2^, Head positioning ^e^	• No changes observed between subgroups in measures of posturography with eyes open or closed.
Robinson, 1987 (USA) [169]	SACT	9 (−-)	Adults (20–40), chronic LBP, unilateral decreased interarticular mobility of SI joint	Spinal manipulation of the sacroiliac joint	Single manipulation	–	–	Gait symmetry^1^	• Gait symmetry data showed trends toward improvement between measures taken at baseline and study completion (χ^2^ = 13.1).
Strunk, 2009 (USA) [166]	SACT	19 (70 median)	Adults (40+) with recurrent dizziness (self-reported) with neck pain	Multimodal chiropractic (SM, flexion distraction, soft tissue therapy, heat)	2x weekly, over 8 weeks (16 sessions total)	–	–	DHI, SF-BBS, NDI, FABQ	Descriptive statistics reported from baseline to study completion: • Median change in DHI score of 7. 3 participants showed clinically significant improvements in DHI scores from baseline to visit 16. 4 additional participants improved scores. • Mean change in SF-BBS score of 3 recorded from the 15 patients that performed SF-BBS. 7 of these patients showed a 4-point improvement from baseline to week 8.
Vining 2020 (USA) [46]	RCT	109 (30)	Active-duty military personnel with LBP	Multimodal chiropractic (clinical evaluation, HVLA SM, education, self-management advice)	Individualized frequency 4 week duration (mean 5.3 visits)	Wait-list control	4 week duration	Strength, single-leg balance with eyes open and eyes closed, endurance, VAS, RMDQ, PROMIS-29, FABQ	• Significant improvement in single-leg balance with eyes closed in chiropractic group. • No significant improvement seen in single-leg balance with eyes open in chiropractic group (*p* = 0.43).
Ward 2013 (USA) [162]	RCT	11 (28.0)	Healthy adults (18–45), college students, no CMT on the study day	HVLA, superior ilium elongation	Single manipulation	No manipulation, participants with one short leg or no short legs	–	Gait variability, ^f^ joint angles, DS time, stance time	• No significant results to report. • Minor trends seen in the treatment group from baseline to study completion with an identified right short leg: increases in step length and stride length, decreases in right hip angle, and changes in double support time.
Ward 2014 (USA) [163]	RCT	21 (25.0)	Healthy adults (18–45), college students, no CMT on the study day	HVLA, Bilateral SI join manipulation	Single manipulation	No manipulation, participants with one short leg or no short legs	–	Gait variability^6^, joint angles, DS time, stance time	• No changes observed from baseline to study completion in intervention group joint angles and gait parameters.

^a^Gait Symmetry/Asymmetry were assessed using a) all 3 components of ground reaction force, and b) for the magnitudes of the maximum and minimum forces of the 3 components of the ground reaction force

^b^Static Postural Control/Posturography was assessed with a computerized stable force plate, using changes in COP under altered visual conditions (eyes open vs. eyes closed)

^c^Multisensory processing reported using millisecond long flashes of light and simultaneously not reporting sound sensors

^d^Static postural assessment was measured with Shimmer3 sensors in three anatomic locations (occiput, second sacral tubercle, and standing surface) during a static postural task under four conditions (1. floor with eyes open; 2. floor with eyes closed; 3. rocker board with AP direction/sagittal plane; 4. rocker board in ML direction/frontal plane). Sensors collected data regarding translation in AP or ML directions, rotation in pitch and roll, pathlength, range, and sample entropy (SampEn)

^e^Head positioning accuracy reported as the subject’s accuracy in relocating the natural head posture was tested after active cervical movements into left and right rotation and flexion and extension

^f^Gait variability was measured with the VICON system, while participants were walking on a treadmill at 1.5 miles per hour. Measurements included double support time for each leg, stance time on each leg, step length, and stride length

### Intervention and control group characteristics

Interventions are summarized in Table 2. Interventions employed within the scope of chiropractic practice were used in all 21 studies. The most commonly used interventions were SM (high-velocity low-amplitude (HVLA) or low-velocity mobilization) [34, 46, 114, 158–160, 162–170, 173], soft tissue manipulation (myofascial release and stretching) [63, 158, 159, 164–166], and thermal therapies (hot or cold packs) [63, 158, 159, 165, 166]. Two studies combined the chiropractic intervention with supervised rehabilitative exercises (SRE) [27, 161]. Nine studies utilized specific techniques such as bioenergetic synchronization (*n* = 1) [158], Activator II^tm^ instrument assisted manipulation (*n* = 1) [63], flexion-distraction (*n* = 1) [166], or specific joint manipulation such as the extremities (*n* = 2) [171, 172], ilium elongation (*n* = 1) [162], and SI joint mobilization (*n* = 3) [163, 168, 169].

Intervention duration ranged from a single session to 20 sessions delivered over 12 weeks. Individual intervention sessions ranged from 4 to 30 minutes, and the frequency of sessions varied between 1 to 3 times per week. Six studies adjusted the frequency and quantity of the SM based on individual needs of participants [34, 46, 114, 161, 164, 174].

Thirteen out of 21 studies used comparison groups in their design. Three studies compared SM with an inactive or waitlist control group [46, 162, 163]. Four used home-based balance exercises or a mix of SM and exercise [158–161], and one compared a short-duration of these combined treatments to a long-duration [174]. One study used sham manipulation [63], and one used usual care as their comparison groups [114]. Finally, three studies used a crossover design; one used SM in combination with facet nerve blockade [170] and two used upper extremity and lower extremity manipulation in alternating order [171, 172].

#### Outcome measures

Findings of the included studies were organized into 3 categories within the following text with respect to their outcomes, which included falls (*n* = 4) [114, 158, 159, 174], balance (*n* = 11) [34, 46, 63, 158, 159, 164–166, 170–172], and gait (*n* = 10) [63, 114, 160–163, 167–169, 173]. These outcomes were considered even when they were not identified as the primary outcome of the study by the authors. Some studies included more than one category of outcomes (*n* = 5) [158, 159, 164, 170, 174].

Four studies reported frequency of self-reported falls; three measured falls prospectively as a secondary outcome measure or as part of adverse events monitoring [114, 158, 159, 174], and one observational study used self-reported falls as a covariate for a separate analysis on neck pain [34]. Clinical measures of balance included the Berg Balance Scale (BBS) (*n* = 4) [34, 158, 159, 165] and short form (SF)-BBS (*n* = 1) [166], measures of center of pressure (*n* = 1) [34], posturography for standing sway/stability (*n* = 4) [34, 170–172], single leg balance assessments (*n* = 4) [46, 158, 159, 165], Activities-specific Balance Confidence Scale (*n* = 1) [34], DHI (*n* = 5) [63, 158, 159, 165, 166], and vertigo severity (*n* = 1) [164]. Measures of gait or mobility included gait symmetry (*n* = 4) [160, 167–169], Timed Up and Go (TUG) (*n* = 2) [63, 161], stepping reaction time (*n* = 1) [114], double support time (*n* = 2) [162, 163], and gait variability (*n* = 2) [162, 163].

#### Adverse events

Ten of the 21 studies provided limited descriptions of their AE monitoring protocols or procedures [46, 63, 114, 158, 159, 161, 165, 166, 172, 174]. All of these reported AEs related to the provided interventions or study procedure including muscle soreness, neck pain, back pain, stiffness, dizziness, and headaches, which resolved within a range of 24-72 h post-manipulation. Only one study reporting AEs mentioned a serious AE that was unrelated to the study intervention [161].

#### Risk of bias assessment

**Fig. 3:**
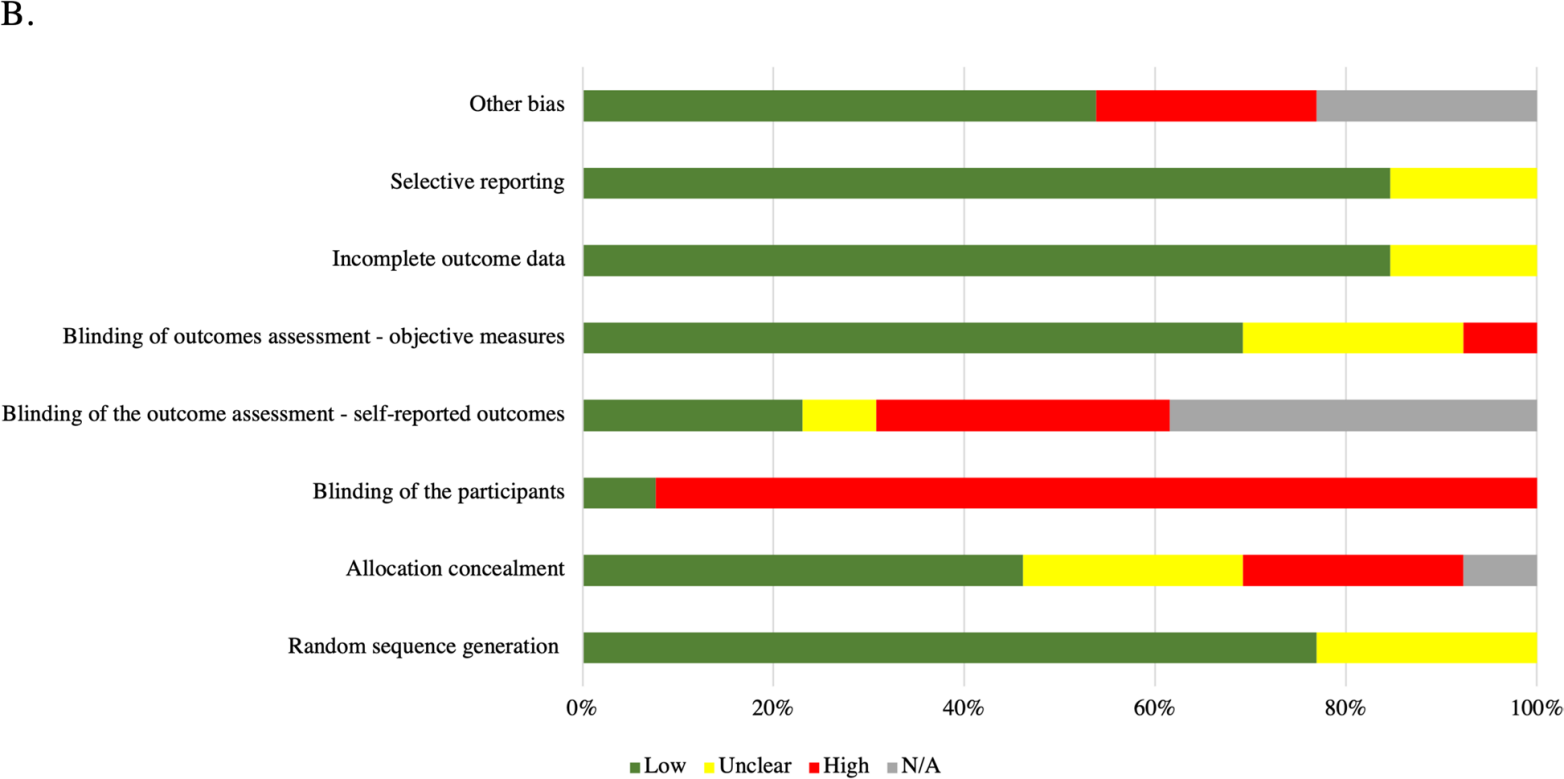
Pooled risk of bias across the 10 RCTS and 3 crossover studies and summarized for each of the 8 reviewed categories

Ten RCTs and three cross-over studies were assessed for risk of bias (Table 3 and Fig. 3). Randomization generation procedures were described in 10 (77%) studies; of those none had a high risk of bias. Low risk of bias was present in 6 studies (46%) for the allocation concealment and only in 1 (8%) for participants’ blinding. 5 studies (38%) did not collect any self-reported outcomes, while the remaining studies had differing risks of bias in this domain with 3 (23%) reporting low risk of bias, 1 (8%) reporting unclear risk of bias, and 4 (31%) reporting high risk of bias. Risk of bias associated with objective measures was low for 9 studies (69%), unclear for 3 (23%), and high for 1 (8%). 11 studies (85%) had low risk of bias for incomplete outcome data and selective reporting, while for the remaining 2 studies (15%) the risk of bias was unclear. High risk for other bias was present in 3 studies (23%), while it was unavailable in 3 (23%) and low in 7 (54%).

**Table 3:** Results from risk of bias assessment for RCTs (*n* = 13) and crossover studies (*n* = 3)

Author	Random sequence generation	Allocation concealment	Blinding of the participants	Blinding of outcome assessment - self-reported outcomes	Blinding of outcomes assessment - objective measures	Incomplete outcome data	Selective reporting	Other bias
***Hawk-2007*** [158]	**+**	**?**	**–**	**–**	**?**	**+**	**+**	**+**
***Hawk-2009-Pilot*** [159, 165]	**?**	**NA**	**–**	**–**	**?**	**?**	**+**	**N/A**
***Herzog-1991*** [160]	**?**	**–**	**–**	**–**	**–**	**+**	**+**	**–**
***Holt-2016*** [114]	**+**	**+**	**–**	**?**	**+**	**+**	**+**	**N/A**
***Kendall-2018*** [63]	**+**	**?**	**+**	**+**	**+**	**+**	**+**	**+**
***Maiers − 2014*** [161]	**+**	**+**	**–**	**+**	+	**+**	**+**	?
***Maiers-2018*** [27]	**+**	**+**	**–**	**+**	+	**?**	**?**	?
***Malaya-2020*** [171]	**+**	**+**	**–**	**N/A**	+	**+**	**+**	+
***Malaya-2021*** [172]	**+**	**?**	**–**	**N/A**	+	**+**	**+**	+
***Palgrem-2009***	**?**	**+**	**–**	**N/A**	**+**	**+**	**+**	**N/A**
***Vining-2020*** [46]	**+**	**+**	**–**	**–**	**+**	**+**	**+**	**+**
***Ward-2013*** [162]	**+**	**–**	**–**	**N/A**	**+**	**+**	**?**	**–**
***Ward-2014*** [133]	**+**	**–**	**–**	**N/A**	**?**	**+**	**+**	**–**

Bias assessment for the RCTs and crossover studies identified through the systematic search. Risk of bias assessment with 8 categories for each individual study

##### Summary of main findings

Impact of Chiropractic Care on Falls, Balance, and Gait.

### Direct measures of falls

Four studies prospectively measured the impact of a chiropractic intervention on fall frequency [114, 158, 159, 174]. Maiers and colleagues evaluated falls as a secondary outcome in a RCT assessing the effectiveness of a long-term combined chiropractic and exercise intervention (36 weeks) compared to a short-term treatment plan consisting of the same combined interventions (12 weeks) for spinal disability (*n* = 182) [174]. Fall incidence was assessed through self-report and direct questioning by the study staff at a clinical encounter every 12 weeks and collected for a 78-week period. In the short-term treatment group, the proportions of fallers reported at each 12-week measure ranged from 6 to 13%; in the long-term treatment group, values ranged from 10 to 13%. Between group differences were not statistically different at any measurement time, and within group changes were not reported for the falls data.

Two small RCTs that used multimodal chiropractic care as an intervention also reported on fall frequency. In 2007, Hawk and colleagues randomly assigned patients (*n* = 11) to two groups that received either multimodal chiropractic care or supervised exercise twice weekly for 8 weeks total [158]. Data on fall frequency and severity was collected at an intake interview section at each appointment, and during an exit interview following study completion. No obvious trends in fall frequency were observed; no statistical analyses were performed. A follow-up study by the same group compared three groups (*n* = 34)–– short-term multimodal chiropractic care (group 1), long-term multimodal chiropractic care (group 2), and home based exercise (group 3) [159]. Frequency of falls were collected through direct questioning at each chiropractic visit and multiple outcome assessments over a period of 1 year. Group one, two and three had six, nine, and zero reported falls, respectively; no statistical analyses or descriptive statistics were reported. Lastly, an RCT (*n* = 60) by Holt and colleagues documented falls as part of adverse events monitoring, reporting seven total falls over the 13-month study period; five in the control group and two in the intervention group [114]. No statistical analyses were reported.

One other study by Holt and colleagues ascertained the history of falls retrospectively from 101 older (mean age 72 y) chiropractic patients using structured interviews, and reported that 34.6% experienced a fall in the previous year; however, no attempt was made to evaluate whether fall history was associated with any aspect of the received care [34].

### Clinical measures of balance

Various measures of balance were collected across 11 studies (total *n* = 391) [34, 46, 63, 158, 159, 164–166, 170–172]. Five studies used BBS or SF-BBS to assess balance characteristics among the study participants, with lower scores indicating a greater risk of falling [34, 158, 159, 165, 166]. A small RCT (*n* = 11) by Hawk and colleagues reported a trend towards improvements in the BBS scores (diminished fall risk) following 8 weeks of multimodal chiropractic care [158], but no descriptive or inferential statistical analysis was used in this study. Similar descriptive trends were reported in another small pilot RCT (*n* = 34) conducted by the same group [159]. Another pre-post single arm study (*n* = 21) limited to descriptive statistics reported that 7 of the 15 patients over the age of 50 treated with multimodal chiropractic care, attained at least a 4-point improvement in the SF-BBS over the 8-week study period, suggesting a possible reduction in fall risk [166]. In another small (*n* = 14) single arm clinical trial assessing multimodal chiropractic care on older adults, findings were limited to descriptive statistics and suggest SF-BBS was largely unchanged after 16 visits [165]. Finally, an observational study (*n* = 101) described, on average, relatively high BBS scores amongst participating chiropractic patients [34].

Four eligible studies used single leg standing outcomes to assess the balance among the participants who received multimodal chiropractic care [46, 158, 159, 165]. One study utilized a single-leg protocol with eyes open and eyes closed, timed with floor sensors (*n* = 109) [46]. Findings from this study indicated statistically significant and favorable results with eyes closed in the group receiving chiropractic care vs. the control group (*p* = 0.01), but no difference in single-leg balance with eyes open (*p* = 0.43). The remaining three studies used the one-leg standing test (OLST) including two RCT’s (*n* < 20 per group) and one single arm clinical trial (*n* = 14). Studies assessed varying frequencies of multimodal chiropractic care. None of these three studies used formal inferential statistics to assess the change in OLST over time. Descriptive summaries of findings suggest no consistent or clinically meaningful changes in OLST scores over the study period.

Another 4 studies used various measures of posturography and standing stability to assess balance [34, 170–172]. One small crossover study (*n* = 6) reported outcomes of posturography, which suggested no significant trends either on anteroposterior (AP) or mediolateral (ML) axis over the study periods [170]. In an observational study of elderly chiropractic patients (*n* = 101), posturographic stability measures including center of pressure and vertical force, were collected from 60 participants standing on a force plate for 20s with their eyes closed, and large impairments were observed [34]. Two crossover RCT studies utilized sensors that gathered outcomes of AP and ML translation, rotation (pitch and roll), pathlength, range, and Sample Entropy (SampEn) from three different anatomic locations (occiput, second sacral tubercle, and the surface participants stood on) during static postural assessments before and after chiropractic manipulation of the extremities [171, 172]. One of these (*n* = 24) reported significant improvements from the sensors at the second sacral tubercle and surface during the AP rocker board condition following upper extremity manipulation (both path *p* < 0.05). Significant improvements were also reported after lower extremity manipulation from the same sensor location and during the same surface condition (SampEn *p* < 0.05). Additionally, greater improvements were reported in range (*p* < 0.05) from the second sacral tubercle sensor in the upper extremity manipulation group when compared to the lower extremity manipulation group. The second study (*n* = 23) followed a similar design, but recorded outcomes related to center of pressure (COP) from force plates rather than from different surface conditions. A reduction of pathlength in the ML COP was observed after both upper and lower extremity manipulation (*p* = 0.005).

Five studies collected data on dizziness using the DHI (*n* = 104) with mixed and inconclusive results [63, 158, 159, 165, 166]. An RCT by Hawk (*n* = 34) showed significant improvements from baseline DHI scores in the two intervention groups receiving different durations of multimodal chiropractic care compared to one group completing balance exercises [159]. Another RCT (*n* = 24) by Kendall and colleagues showed DHI scores with over 10 points decrease in both the intervention (4 weeks of multimodal chiropractic care including instrument assisted spinal manipulation, massage, heat, and exercise) and sham intervention groups [63]. A third RCT (*n* = 11) and a single arm clinical trial (*n* = 21) utilized intervention groups which received 16 sessions of multimodal chiropractic care. Both trials showed little to no improvement in DHI scores [158, 166]. An additional small single arm clinical trial (*n* = 14) utilizing the same intervention showed mixed results regarding participant’s DHI scores following the intervention period [165]. Lastly, a single arm clinical trial (*n* = 15) completed by Bracher and colleagues measured the severity of cervical vertigo symptoms following a novel combined treatment intervention including spinal manipulation, electrotherapy, medication and biofeedback exercise. Results showed that 12 of the study participants experienced either remission or improvement of cervical vertigo over the course of treatment; however, the results varied by the measured severity of the subjects’ vertigo [164].

### Gait

Ten studies collected data on mobility and gait health parameters (total *n* = 434) [63, 114, 160–163, 167–169, 173]. Two studies used TUG tests to assess mobility in older adults (*n* = 265). Maiers and colleagues reported on TUG test times in two experimental groups, one receiving multimodal chiropractic combined with home exercise and one receiving SRE combined with home exercise, as well as one control group which completed only home exercise [161]. Results showed no significant between group differences in TUG test time measures taken at 12 weeks compared to baseline [161]. Measures from two-arm RCT by Kendall and colleagues (*n* = 24) reported small TUG test time improvements in the group receiving chiropractic between baseline and 4-week completion, however no statistical analysis was performed [63].

In an RCT (*n* = 60) comparing individualized chiropractic treatment vs ‘usual care’ in community-dwelling older adults [114], chiropractic treatment lead to improvements in choice stepping reaction time (*p* < 0.05) and ankle joint position sense (*p* < 0.05) compared to the control group [114]. Two small RCTs (*n* = 11) completed by Ward and colleagues measured the impact of bilateral SI joint manipulation on gait variability (defined as approximate entropy of the hip, knee, and ankle joint angles over multiple gait cycles) captured with a VICON motion analysis system among individuals with a leg length inequality [162, 163]. Neither of these studies reported improvements in gait variability measures following the intervention [162, 163].

A 2-week single arm clinical trial (*n* = 11) by Herzog and colleagues utilizing force platform analysis, resulted in a statistically significant difference between the distribution of gait loading forces (i.e., vertical and mediolateral) post SI joint manipulation [167]. Reduction in pain on visual analogue scale was also associated with improved gait parameters and decreased ground reaction forces in AP and ML directions, indicative of decreased gait asymmetry [167]. However, the results from follow-up studies by Herzog and colleagues were not consistent. In a single arm clinical trial (*n* = 11) no changes in gait asymmetry were found following a single manipulation session [168], while a 4-week RCT (*n* = 37) completed afterwards showed a significant improvement on all measured ground reaction forces in the spinal manipulation group compared to a physiotherapy ‘Back School’ intervention [160, 168].

Another small single arm clinical trial (*n* = 9) evaluated the effect of a single manipulation by utilizing force platform analysis which measured temporal and kinetic variables, and showed SI joint manipulation has a tendency to reduce gait asymmetries assessed through a symmetry index [169]. Conversely, a biomechanical analysis of gait conducted in a 5-week case series (*n* = 10) focused on the use of mechanical and manually assisted SM for SI joint mobility, showed no differences in gait or gait asymmetry in study subjects pre- and post- treatment [173].

## Discussion

The initial narrative section of this paper on the impact of chiropractic care and its individual component modalities on multiple fall-related risk factors supports the idea that chiropractic care targeting musculoskeletal strength and flexibility, pain, proprioception, vestibular function, polypharmacy, and education could be a promising intervention for reducing falls and improving balance and mobility in older adults. However, our embedded formal systematic review did not identify a single clinical trial designed and adequately powered to directly evaluate the long-term impact of chiropractic care on the prevalence of falls in older adults. Moreover, the majority of studies evaluating the impact of chiropractic care on clinical measures of balance and mobility known to be associated with fall risk, were small, statistically underpowered, of low methodological quality, and typically did not target older adults at risk for falls. Thus, while the potential seems to be clear, our findings both highlight a significant gap in the evidence base for the use of chiropractic care for fall prevention, and underscore the need for developing future directions for research evaluating both the effectiveness of chiropractic care for reducing falls and fall-related injuries in older adults. Our findings also point to the need for research aimed at understanding the mechanistic processes through which the individual and combined components of chiropractic care impact fall-related risk, which could help refine and target intervention to meet the needs of populations with specific risk factors and related comorbidities.

Four clinical trials prospectively measured the impact of elements of chiropractic care on falls. The largest and most methodologically sound study was a 2 arm RCT by Maiers and colleagues that compared two durations of combined chiropractic care with SRE, and collected falls data via self-report [174]. Findings from this study, however, are limited by lack of a non-intervention control group and lack of information regarding the statistical power required to detect between group differences in falls (which were defined a priori as a secondary outcome). Variability in how and when falls data were collected, and complete reliance on self-report and recall, with no method to adjudicate falls, also limit the rigor of this and all studies in this review with respect to evaluating falls [175]. A second study was also not designed or powered for detecting changes in fall frequency following an intervention, with information only collected as part of adverse events monitoring [114]. Additionally, both studies completed by Hawk and colleagues were also underpowered to detect falls and data were not statistically analyzed [158, 159]. Collectively, these studies illustrate the challenges researchers face in quantifying fall frequency in the context of clinical trials. Recent trials on multifactorial fall prevention programs offer some guidance for improving the rigor associated with estimating fall frequency including combining the use of patient completed fall logs with systematic phone or in-person queries [176–180], or adjudicating logs with reports embedded within medical records (e.g., emergency department visits or radiographic records [181–183]. Other approaches that leverage emerging technologies include the use of wearable sensors or camera-based home surveillance for long-term continuous monitoring of high-risk populations [184, 185].

The most commonly assessed outcome measures in the studies identified by our formal systematic review were related to measures of balance and gait. With respect to balance, five studies collected BBS (or SF-BBS) scores [34, 158, 159, 165, 166], which in recent reviews has shown conflicting agreement for its suitability in predicting falls [186, 187]. Five of the included studies collected DHI scores [63, 158, 159, 165, 166], which is a widely accepted clinical measure for assessing the impact of dizziness on quality of life and activities of daily living [188, 189]. All of the studies reporting BBS (or SF-BBS) and DHI scores were small and used descriptive statistics to report their data, therefore no conclusions can be drawn on the impact chiropractic care may have on either of these outcomes. Four studies collected posturography measures for standing sway or stability using ground reaction forces from force plates or body sensors [34, 170–172], and an additional four collected measures of gait symmetry [160, 167–169]. While each of these clinical balance outcomes evaluated has shown some links to fall risk [154, 187, 190–192], there remains much debate about the best measures of balance for predicting falls [193–195]. Future studies should consider recently validated outcome measures, which take into account the dynamic conditions of balance such as the Balance Evaluation Systems Test (BEST), or miniBEST, which have shown promise in identifying and differentiating balance and gait deficits [196–198] as well as correlations to falls in the older population [199, 200]. Similarly, multiple characteristics of gait, including speed and stride time variability, have also been associated with fall risk [154]. Unfortunately, most of the identified studies that evaluated gait were small and did not include statistical analyses, limiting their contribution to our understanding of chiropractic care on mobility and fall prevention. Well-designed studies evaluating the impact of chiropractic care on key parameters of gait health in at risk older adults should be a priority area for research. In addition to outcomes associated with gait health informing practical issues of mobility and functional ability to engage in everyday activities of daily living, they also provide insight into the biomechanical and neurophysiological processes underlying fall risk. There is also growing evidence that gait assessed outside the laboratory using wearable sensors, during activities of daily living, may be particularly helpful in predicting falls, and thus future studies should also consider outcomes assessing gait under daily living conditions [201, 202].

### Proposed strategies for building an evidence base to inform the role of chiropractic in fall prevention

Below we propose several strategies that may be used to begin addressing the evidence gap related to chiropractic care and fall prevention. While ultimately, fully powered multi-site RCTs will provide the least biased and most rigorous source of evidence, given both the cost of such trials as well as the current paucity of studies to inform their design, large scale RCTs may not be the most effective strategy for beginning to build an evidence base. Rather, initial approaches which aim to build foundational knowledge to inform such trials, including the leveraging of existing large healthcare databases for observational or retrospective analyses, pilot clinical trials, and mechanistic studies to better understand the effects of specific components of multimodal chiropractic care on known fall risk factors are likely to yield the most pragmatic and initially informative evidence.

#### Observational studies leveraging existing databases

One practical first step to better understanding the association between chiropractic care and fall risk would be to leverage data from existing large databases available through managed care facilities, health claims data, and clinical systems such as the VA. Previous studies utilizing such databases, for example, have determined that chiropractic care is associated with reduced likelihood of adverse drug events [203] and filling opioid prescriptions [204], as well as determining correlations between chiropractic patient characteristics (i.e. gender, age, body mass index) and clinical outcomes such as pain and disability [205, 206]. Such databases could be leveraged to characterize the relationship between incidence of falls related outcomes and prior and/or future exposure to chiropractic care. For example, retrospective analyses could include comparisons between chiropractic and non-chiropractic patients in terms of frequency of reported falls, fall-related injuries, and medical costs associated with falls. Importantly, large databases could include invaluable information related to comorbidities (e.g., pain conditions, depression), risk factors for falls (e.g., polypharmacy), and multiple sociodemographic factors known to impact fall risk. Integrating these variables into thoughtful, pre-specified analyses could lead to more focused, population specific hypotheses to evaluate in clinical trials. Such approaches could rely on well-established statistical approaches, including multivariate modeling, propensity score matching, and machine learning [207–211].

One potential challenge in retrospective studies using databases could be the availability of consistent and reliable data on falls. Prior studies suggest that this information is not always clearly coded in an electronic medical record but may be obtained through more innovative methods such as natural language processing or machine learning algorithms applied to medical notes within electronic health records. Several approaches have been shown to be successful utilizing International Classification of Disease codes in medical claims data to determine risk of opioid prescription for chiropractic patients [141, 181, 212–216] and developing sophisticated algorithms for the analysis of falls and related outcomes such as injuries and cost [181]. Chiropractic health care services at the VA show promise in this regard due to both the increasing number of chiropractors nationwide in their complete health care system and previous research which has extracted data on falls utilizing the above-described methods [217].

Another alternative observational study approach would be a non-randomized prospective cohort study with a non-exposed group control. For example, patients in a large Health Maintenance Organization who are screened for high fall risk and enrolled in chiropractic care could be longitudinally and systematically monitored for fall related outcomes and compared to equivalent patients not receiving chiropractic care. Advantages to this approach include interventions reflecting real-world care, reduced effort in patient recruitment, and higher enrollment rates (due to certainty of allocation). Limitations to this approach include difficulty in matching groups with respect to potential confounders even with large sample sizes. As with retrospective observational studies, non-randomized prospective trials may be useful in the early stages of research by informing design features of future randomized trials, such as most responsive population, dosage for exposure, and specific components of multimodal interventions.

#### Randomized controlled trials

An essential step to further progress in building an evidence base for chiropractic services in the realm of fall prevention would be the initiation of large-scale prospective RCTs specifically targeting adults at risk for falling, fully-powered to detect changes in frequency and severity of falls. Based on both the general literature on the efficacy of multifactorial interventions for falls [201], as well as the effectiveness of chiropractic care for many musculoskeletal conditions, the evaluated chiropractic interventions should be multimodal as opposed to isolated components (e.g. SM alone). Promising interventions provided by chiropractors should likely include combinations of SM, myofascial therapies, supervised and prescriptive exercise, and self-management strategies. Pragmatic designs might be used to evaluate the relative effectiveness of different combinations of these interventions, different dosages, and different strategies for implementation (e.g., combining in person care with virtual education or exercise training). Comparisons may also be made to existing programs recommended by the National Council on Aging, such as Tai Chi or Otago, or usual medical care, employing comparative effectiveness designs, or equivalency or non-inferiority designs [218, 219]. As noted above, advances in digital medicine regarding the use of wearable devices have recently established the relationship between everyday gait and injurious falls in the older population, providing an alternative approach to objectively assessing gait health and fall risk following well-defined exposures [202, 220]. Finally, these studies could be combined with data regarding medical utilization and costs to evaluate the cost effectiveness of these interventions.

As with every large-scale trial, smaller scale feasibility and pilot trials should be conducted to inform final study features including recruitment and retention procedures, fidelity of intervention delivery, selection of primary and secondary outcomes and protocols for outcomes assessment, infrastructure for data management and ethical oversight across multiple sites. Consolidated Standards of Reporting Trials (CONSORT) and other guidelines for the design and reporting of pilot clinical trials are now widely available [221, 222].

Additional consideration could be given to experimentally evaluating the benefits of delivering existing validated fall preventions resources to chiropractic providers in order to better identify fall risks within their older adult patients. Existing high-quality educational resources which recommend multifactorial approaches to fall prevention include the CDC’s Stopping Elderly Accidents, Deaths & Injuries (STEADI) Older Adult Fall Prevention [18], or those endorsed by the United States Preventive Services Task Force [223], could be administered to and adopted by chiropractors in private clinical practice and within large healthcare systems. Once identified, high-risk fallers could be triaged to specific evidence-based interventions or protocols, or ongoing trials evaluating novel therapeutic approaches.

#### Mechanistic studies

Elucidating the mechanism underlying any therapeutic intervention is important, as plausible mechanistic models are considered to be part of the totality of evidence for any modality. Chiropractic care’s multimodal nature and heterogeneity in its delivery poses challenges to simple, single-factor, cause-and-effect reductionist models. As is emphasized in the narrative part of our review, the impact of a chiropractic care intervention on fall risk is likely to result from changes in multiple physiological processes, individually, additively, and synergistically. For example, observed changes in falls following a multimodal intervention could be results of improvements in lower extremity strength and flexibility, enhanced proprioception, and reduced pain and associated executive function distraction. These changes could lead to behavioral changes (e.g., reduced fear of falling and enhanced exercise self-efficacy) or underlying changes in brain network dynamics, which might feedback and further alter physiological process previously mentioned. For these reasons, a strong case can be made for focusing limited resources on pragmatic trials that evaluate overall effectiveness of chiropractic intervention.

Despite complexity and heterogeneity related challenges inherent to chiropractic care, mechanistic research can be advanced in three complementary ways. First, specific cause and effect hypotheses could be tested with carefully designed efficacy trials, extending some of the studies cited in this review. For example, RCTs could evaluate the impact of a well-defined and systemically delivered SM protocol on pelvis or lower extremity electromyography (EMG) dynamics and resulting biomechanics of gait known to contribute to balance and postural control. Attention or sham manipulation experimental controls could be utilized, as can dose escalation designs, to infer cause and effect. Recent studies have used such designs to probe the impact of short-term SM on brain neural networks [224, 225].

Another approach to inform a mechanistic understanding could be based on traditional factorial designs, or newer strategies including Multiphase Optimization Strategy (MOST) and the Sequential Multiple Assignment Randomized Trial (SMART) designs. Such approaches can inform interactions between therapeutic components on both specific physiological outcomes (e.g., joint biomechanics) or more synthetic clinical outcomes (frequency of falls). Examples of factorial designs being employed in chiropractic research includes evaluating the individual and combined effects SM and prescribed exercise, or SM and self-care on musculoskeletal pain [47, 48, 86, 146, 148, 161, 174].

A third and complementary approach would be to embed a variety of carefully chosen physiological markers into pragmatic trials to test specific secondary hypotheses regarding their association with falls. For example, laboratory measures ranging from brain function assessed during movement tasks using functional magnetic resonance imaging (fMRI) to conventional markers of gait health (e.g., gait initiation, speed, stride time variability, dual task gait performance) could be assessed longitudinally over the course of an intervention. Multivariate analyses, including tools such as structural equation modeling could be employed to inform or test causal hypotheses linking specific physiological or biomechanical processes to clinical outcomes of interest (e.g., fall frequency or severity). As noted above, recent advances in wearable technology could be used to assess a diverse array of mobility markers during everyday living over extended periods of time. Observational studies using machine learning show promise informing the relationship between these physiologic markers assessed in the real world and fall risk [182, 183, 217], but have not yet been widely used in intervention studies.

### Limitations

There are a number of limitations to this study. First, only studies in the English language were included, the search strategy was developed without formal help from a librarian, and a protocol was not developed or registered a priori. Second, based on the limited evidence to date, only a small number of studies met our inclusion and exclusion criteria. Third, there was a great deal of heterogeneity across the included studies in terms of study design characteristics, methodologic quality, demographics of participants, interventions, outcomes, and settings, excluding the possibility of quantitative synthesis with meta-analysis, and significantly constraining narrative synthesis. Of note, the majority of included studies were small pilot studies, with overall low methodological quality. Collectively, this heterogeneity and low methodological quality limits the conclusions that can be drawn from these data. Fourth, our inclusion criteria targeted studies that included evaluations of components of multimodal chiropractic care (e.g. SM, myofascial therapies), as delivered by a DC. Some of these modalities are also delivered by other professions (e.g., osteopath, physical therapist), and because of the limits of our search strategy, our findings cannot distinguish chiropractic-specific impacts of the included interventions compared to the impacts of these interventions as delivered by other professions.

## Conclusions

Falls in the older population represent a large and growing public health issue. Based first on principle, multimodal chiropractic care shows promise in contributing positively to fall prevention efforts. In addition, the chiropractic profession is well positioned for implementation on a wide scale, however, to date limited evidence is available. Through a well-coordinated set of observational, mechanistic and randomized-controlled studies, this evidence gap can be filled and the potential of multimodal chiropractic care can be evaluated. Future research on falls and mobility represents both an exciting area of contribution for the chiropractic profession and a critical topic for public health.

## Supplementary Information

**Additional file 1.**

**Additional file 2.**

## Data Availability

All data generated or analyzed during this study are included in this published article and its supplementary information files.

## Abbreviations

ABCs: Activities-Specific Balance Confidence Scale
AE: Adverse Events
AP: Anteroposterior
APA: Anticipatory Postural Adjustments
BBS: Berg Balance Scale
BEST: Balance Evaluation Systems Test
CDC: Centers for Disease Control and Prevention
COG: Center of Gravity
CONSORT: Consolidated Standards of Reporting Trials
COP: Center of Pressure
DC: Doctor of Chiropractic
DHI: Dizziness Handicap Inventory
DS time: Double Support time
DSP%: Double Stance Phase Percentage
EMG: Electromyography
EQ-5D: European Quality of Life Five Dimensional Instrument
FABQ: Fear Avoidance Beliefs Questionnaire
FES-I: Falls Efficacy Scale International
fMRI: Functional Magnetic Resonance Imaging
GDS: Geriatrics Depression Scale
GRF: Ground Reaction Forces
HVLA: High-Velocity Low-Amplitude
IASTM: Instrument Assisted Soft Tissue Manipulation
LBP: Low Back Pain
ML: Mediolateral
MMSE: Mini-Mental State Examination
MOST: Multiphase Optimization Strategy
NDI: Neck Disability Index
ODI: Oswestry Disability Index
OLST: One-Leg Stance Test
PDI: Pain Disability Index
PNF: Proprioceptive Neuromuscular Facilitation
PRISMA: Preferred Items Reporting for Systematic Reviews and Meta-Analysis
PROMIS-29: Patient-Reported Outcomes Measurement Information System
RCT: Randomized Controlled Trial
RMDQ: Roland Morris Disability Questionnaire
ROM: Range Of Motion
SACT: Single Arm Clinical Trial
SF-36: 36 Item Short Form Survey
SF-BBS: Short-Form Berg Balance Scale
SI: Sacroiliac
SM: Spinal Manipulation
SMART: Sequential Multiple Assignment Randomized Trial
SPPB: Short Physical Performance Battery
SRE: Supervised Rehabilitative Exercise
STEADI: Stopping Elderly Accidents, Deaths & Injuries
TENS: Transcutaneous Electrical Nerve Stimulation
TO: Toe-Off
TSK: Tampa Scale of Kinesiophobia
TUG: Timed Up and Go
VA: United States Department of Veterans Affairs
VAS: Visualized Analog Scale
